# Correlation of prognostic values of IL-6 and PCT levels with the severity of pneumonia caused by *Mycoplasma pneumoniae* in children

**DOI:** 10.12669/pjms.41.5.10448

**Published:** 2025-05

**Authors:** Xinxin Bao, Qiuhong Wang, Haiying Geng, Xiaohua Yuan

**Affiliations:** 1 Xinxin Bao Department of Clinical Laboratory, Affiliated Maternity and Child Health Care Hospital of Nantong University, Nantong, Jiangsu 226001, China; 2 Qiuhong Wang Department of Clinical Laboratory, Affiliated Maternity and Child Health Care Hospital of Nantong University, Nantong, Jiangsu 226001, China; 3 Haiying Geng Department of Clinical Laboratory, Affiliated Maternity and Child Health Care Hospital of Nantong University, Nantong, Jiangsu 226001, China; 4 Xiaohua Yuan Department of Clinical Laboratory, Affiliated Maternity and Child Health Care Hospital of Nantong University, Nantong, Jiangsu 226001, China

**Keywords:** Interleukin 6, Lung function, Mycoplasma pneumoniae pneumonia, Procalcitonin, severity, Prognostic predictions

## Abstract

**Objective:**

To explore the correlation of interleukin 6 (IL-6) and procalcitonin (PCT) with severity of *Mycoplasma pneumoniae pneumonia* (MPP) in children and their prognostic value.

**Methods:**

One hundred twenty children with MPP and 100 healthy children who underwent physical examination at the Clinical Laboratory Department of Nantong University Maternal and Child Health Hospital from November 2023 to December 2023 were selected as the study subjects, and were divided into the experimental group and the control group. The experimental group was divided into mild MPP group (n=70 ) and severe MPP group (n=50 ) according to the severity of the disease. According to the condition after one course of treatment, separated MPP children into good prognosis group (n=96) and poor prognosis group (n=24). The IL-6 and PCT levels of the experimental/control group before treatment and the good/bad prognosis group after treatment were compared, respectively. The lung function of children with MPP before and after treatment was also examined, and the changes of respiratory rate (RR), tidal volume (VT), expiratory time (Te), peak time (TPTEF), and peak volume (VPTEF) were observed. The correlation between pulmonary function and IL-6, PCT in MPP children was analyzed by Pearson analysis. Receiver operation curve was adopted to analyze the predictive value of IL-6, PCT for poor prognosis.

**Results:**

IL-6 and PCT were increased in the severe MPP group compared to the mild MPP group (P<0.001) and tended to decrease in the good prognosis group compared to the poor prognosis group (P<0.001). Fifty-nine of the 70 cases in the good prognosis group were patients with mild MPP, suggesting that patients with mild MPP have a better prognosis. The results of respiratory function showed an increase in RR (P<0.001) and a decrease in VT (P=0.007), TPTEF/Te (P<0.001), VPTEF/VT (P<0.001) in the severe MPP group compared to the mild MPP group. The results of correlation analysis revealed that IL-6, PCT were negatively correlated with VT, TPTEF/Te and VPTEF/VT (P<0.001). ROC curve analysis revealed that IL-6 and PCT co-diagnosis had a better predictive ability for poor prognosis in children with MPP (AUC=0.843).

**Conclusion:**

IL-6 and PCT are positively related to the severity of MPP, the combined detection of IL-6 and PCT has certain clinical usefulness to evaluate and assess prognosis of MPP children.

## INTRODUCTION

Mycoplasma pneumoniae pneumonia (MPP) belongs to an acute lung inflammation caused by mycoplasma pneumoniae (MP) infection, which is frequent in school-age children and young adults, as well as infants.^1^. According to relevant research reports, the common clinical manifestations of MPP include cough, fever, wheezing, cyanosis, nasal congestion, shortness of breath and other symptoms, the lack of specificity in the early manifestations of the disease, resulting in a high early misdiagnosis rate, affecting the early diagnosis and treatment of children, therefore, early diagnosis as well as effective treatment is the key to improve the prognosis of children.^2^.

Currently, the clinical diagnosis of mycoplasma pneumonia mainly relies on mycoplasma culture and mycoplasma antibody detection, but due to technical reasons and early sensitivity limitations, often prolong the time of disease diagnosis in children.^3^. MP has a complex pathogenesis and generally causes respiratory infections. Evidence suggests that inflammatory response, immune response and other manifestations are involved in the pathogenesis of MPP infection. Interleukin 6 (IL-6) and procalcitonin (PCT), as common important indicators reflecting the degree of inflammation, will increase significantly when the body has infection and increase with the progression of MPP.^4^. IL-6 is a multifunctional acute response cytokine.^5^,^6^

In the current research on the pathogenesis of MPP in children, the immune mechanism is recognized by most scholars, and cytokines are believed to be related to the pathogenesis.^7^ When MP infects the body, endogenous inflammatory mediators IL-6 and IL-8 are activated and released, and a large number of other cytokines are released.^8^. IL-6 comes from a wide range of sources, mainly from mononuclear macrophages and activated T cells.^9^ IL-6 has a crucial role in inflammatory response, anti-infection as well as autoimmunity.^10^. As an important cytokine in inflammatory response, IL-6 forms a complex cytokine network with other factors and participates in the pathological process of lung inflammation.^11^. Procalcitonin (PCT) belongs to a novel inflammatory marker found by Assicot et al. in 1993.^12^. Some studies have shown that serum PCT can indicate the observation index of inflammation caused by bacterial infection. PCT is a hormonally inactive precursor of calcitonin with a half-life of 25 to 30 hours.^13^; ^14^. The main source of PCT in vivo under pathological conditions is the liver.^15^. The content of PCT in the serum of healthy people presents very low, and the possibility of detection is very small.^16^. As the severity of the disease increases, so do the levels of procalcitonin. However, after treatment, PCT levels decline rapidly, and testing for this feature can be an indicator of bacterial infection.^17^.

Lung function is mainly used to reflect the severity of MPP in clinical practice. Evidence suggests that when the disease is severe, the immune response and pro-inflammatory factors such as IL-6 are activated in the body, causing alveolar inflammation, which affects the normal physiological function of the lungs, leading to a decline in lung function and respiratory dysfunction.^18^ And Zhu et al. found that the impairment of lung function by organophosphorus garlic ester flame retardants was mediated through the IL6/JAK pathway.^19^. In addition, RR, VT, TPTEF/Te, VPTEF/VT are important indicators for assessing pulmonary function in MPP. After drug treatment of MPP, patients showed significant improvement in IL-6, PCT, and pulmonary function indicators such as FVC, FEV1, and FEV1/FVC.^20^;^21^ Therefore, further study of the correlation between IL-6/PCT and lung function may be able to increase the credibility of IL-6/PCT on the prognosis of MPP.

The aim of this study was to improve the early diagnosis and prognosis assessment of MPP in clinical practice. Therefore, we investigated the correlation between IL-6 and PCT levels and the severity of MPP in children and analyzed the predictive value of IL-6 /PCT on the prognosis of MPP.

## METHODS

This was a retrospective study to investigate the predictive value of IL-6 and PCT on the prognosis of children with MPP. One hundred twenty children with MPP and 100 healthy children who received physical examination in the Clinical Laboratory Department of Nantong University Affiliated Maternity and Child Health Hospital from November 2023 to December 2023 were selected for study subjects.

### Ethical Approval:

Guardians were aware of and consented to the study. This study was approved by the Ethics Committee of our hospital (Y2022027), Date: February 27, 2022.

### Inclusion Criteria for Children with MPP:

- Patients met the diagnostic criteria of MPP.
- Course of disease ≤14 days.
- The guardian of the child volunteered to participate in the experimenter.

### Exclusion criteria:

- Patients with hematological diseases, neurological diseases and autoimmune diseases.
- Patients who had been treated with immunosuppressants or glucocorticoids within one month before enrollment.
- Patients with asthma.

The children with MPP were divided into mild MPP group (n=70) and severe MPP group (n=50) according to the severity of the disease. Children meeting any of the following conditions were included in the severe MPP group: (i) Obvious shortness of breath or cyanosis; (ii) Chest imaging showed multiple lobe involvement or ≥2/3 lung area; (iii) Accompanied by pleural effusion or extrapulmonary complications. (iv) arterial oxygen saturation ≤0.92.

After admission, all MPP children were given intravenous injection of azithromycin (Manufacturer: Hainan Puli Pharmaceutical Co., LTD., Specification: 0.5 g) for treatment, once/d, 10 mg/kg each time, the course of treatment was 1-2 weeks. The children were given water and electrolyte balance and other treatments. Children with severe MPP were additionally treated with aerosol inhalation of budesonide suspension (Manufacturer: Zhengda Tianqing Pharmaceutical Group Co., LTD., Specification: 2 mL: 1 mg) or oral ibuprofen suspension (Manufacturer: Shanghai Johnson Pharmaceutical Co., LTD., Specification: 100 mL: 2 g) treatment. Children with MPP were preferentially treated with macrolides such as azithromycin, and if no significant effect was found and the results of drug sensitivity tests showed resistance, the type of antibiotic was changed, with preference given to macrolides, followed by fluoroquinolines and tetracyclines. Children in the healthy control group did not require any treatment. Discontinue antibiotics when the PCT value is less than 0.25 or when there is an 80% decrease from the peak value.

After one course of treatment for children with MPP, 59 children with mild MPP had a good prognosis and 11 had a poor prognosis. Ten children with severe MPP had a good prognosis and 40 had a poor prognosis. The prognosis was judged on the basis of clinical symptoms, signs and laboratory indicators, i.e., the prognosis was poor if there was no significant decrease in body temperature, worsening of cough and pulmonary symptoms, chest radiographs suggesting absorption or aggravation of pulmonary lesions, or the need to be transferred to a higher level hospital for treatment. The child’s body temperature is normal, cough and asthma symptoms are significantly relieved, and the chest X-ray shows absorption or disappearance of the lung lesion. If one or more of the above criteria are met, the prognosis is good.

Venous blood of external elbow was collected from all MPP children in the morning of the next day after admission. Healthy children underwent fasting source physical examination, venous blood was collected first, centrifuged at 3500 r/min, and serum was separated. IL-6 and PCT levels were examined by using quantum dots-based immunofluorescence chromatography (Manufacturer: Shenzhen Kingfocus Biomedical Engineering Co., Ltd.). Compare IL-6 and PCT levels between healthy children, children with mild MPP and children with severe MPP. Compare IL-6 and PCT levels between the group with a good prognosis and the group with a poor prognosis after treatment.

On the day of examination, the nasal secretions were cleaned up, the body mass of the child was accurately measured, and a suitable mask was selected to cover the face to ensure no air leakage. Parameters of the pulmonary function instrument were adjusted, and respiratory rate (RR), tidal volume (VT), expiratory time (Te), peak time (TPTEF), peak volume (VPTEF) of children were observed, and TPTEF/Te and VPTEF/VT were calculated.

### Statical analysis:

SPSS 22.0 software was adopted to process the data. The measurement data were exhibited as (x±s) and analyzed by t test. The counting data were expressed as n (%) and analyzed by χ^2^ test. The correlation between IL-6, PCT and respiratory function (RR, VT, TPTEF/Te, VPTEF/VT) of children with MPP was analysed by Pearson correlation analysis. Receiver operation curve (ROC)was implemented to analyze the predictive value of IL-6, PCT for poor prognosis in MPP children. A p-Value <0.05 was considered statistically significant.

## RESULTS

We compared the general data of the included subjects, where the mild MPP group consisted of 40 boys and 30 girls with a mean age of (4.76±2.21) years and a mean weight of (22.49±1.28) kg. The severe MPP group consisted of 26 boys and 24 girls with a mean age of (4.74±2.16) years and a mean weight of (22.52±1.29) kg. The control group consisted of 55 boys and 45 girls with a mean age of (4.71±2.22) years and a mean weight of (22.56±1.32) kg. There was no difference in the general data of the three groups (P>0.05). The poor prognosis group after MPP treatment consisted of 13 boys and 11 girls with a mean age of (4.75±2.25) years. In the good prognosis group, there were 53 boys and 43 girls with a mean age of (4.76±2.18) years. There was no difference in general data between the two groups (p>0.05).

**Fig.1:**
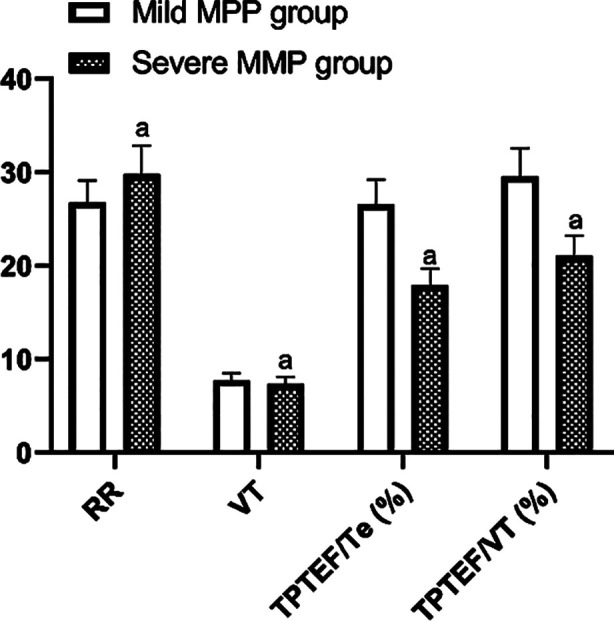
Pulmonary function in children with MPP of different severity. ^a^P<0.05.

**Fig.2:**
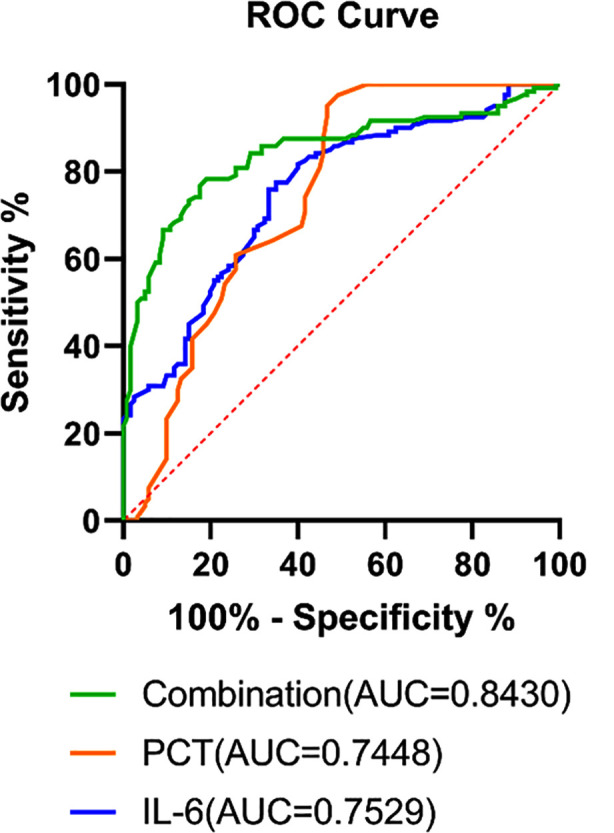
Prognostic value of IL-6, PCT levels alone and in combination in children with MPP.

As shown in Table-I, IL-6 and PCT levels were higher in both the severe MPP group and the mild MPP group than in the control group (P<0.001). What’s more, both IL-6 and PCT levels were increased in the severe MPP group compared with the mild MPP group (P<0.001). This suggests that IL-6 and PCT levels may be associated with the severity of MPP. Lung function is one of the indicators of the severity of MPP, and we compared the lung function of children with mild MPP and severe MPP. The results showed high RR and low VT(P=0.007), TPTEF/Te (P<0.001) and VPTEF/VT(P<0.001) in the severe MPP group compared with the mild MPP group (Fig.1), suggesting that lung function was worse in severe MPP. Therefore, we correlated IL-6 / PCT levels with lung function to further validate the correlation between IL-6 / PCT and MPP severity. In Table-I IL-6 and PCT levels did not correlate with RR (P>0.05), but negatively correlated with VT, TPTEF/Te and VPTEF/VT (P<0.001), indicating that the higher the IL-6 and PCT levels, the worse the lung function. This is further evidence that IL-6 and PCT levels may be positively correlated with the severity of MPP. The prognostic prediction of IL-6 and PCT levels for children with MPP was further explored, and IL-6 and PCT levels tended to decrease in the good prognostic group compared with the poor prognostic group (P<0.001, Table-I).The ROC curve analysis showed that the combined predictive value of IL-6 and PCT for poor prognostic of children with MPP (AUC=0.843) was higher than that of detecting the two individually (P< 0.05, Fig.2).

**Table-I:** Comparison of IL-6 and PCT levels in different subgroups.

Groups	IL-6 (pg/mL)	PCT (ng/mL)
Control group(n=100)	5.45± 0.54	0.026± 0.003
Mild MPP group((n=70)	22.26± 2.32	0.23± 0.02
Severe MMP group(n=50)	46.15± 4.62	0.36± 0.04
***P1-values***	<0.001	<0.001
***P2-values***	<0.001	<0.001
Good prognosis group(n=96)	24.65± 2.46	0.28± 0.03
Poor prognosis group(n=24)	69.02± 7.02	0.52± 0.06
***P3-values***	<0.001	<0.001

P1: Control group versus Mild MPP group; P2: Mild MPP group versus Severe MMP group; P3: Good prognosis group versus Poor prognosis group.

**Table-II:** Correlation analysis of IL-6 together with PCT levels and lung function in children with MPP.

Index	IL-6		PCT	
	r value	P-values	r value	P-values
RR	0.056	0.408	0.134	0.349
VT	-0.741	<0.001	-0.735	<0.001
TPTEF/Te	-0.746	<0.001	-0.739	<0.001
VPTEF/VT	-0.692	<0.001	-0.681	<0.001

## DISCUSSION

In this study, we found that IL-6 and PCT were positively correlated with the severity of MPP, and the combined detection of IL-6 and PCT has some clinical benefits in assessing the prognosis of children with MPP. Pneumonia induced by MP is a common respiratory disease in pediatrics.^22^. With fever and cough as the main symptoms, it is easy to be regarded as a common cold. Without standard treatment, it is easy to damage the heart, lungs, and other organs of patients, and even threaten their lives.^23^. Therefore, timely screening of severe patients and patients with poor prognosis and giving corresponding prevention and treatment measures are very important to save the life and health of patients. Prediction of adverse outcomes in patients with MMP is one of the therapeutic strategies for MPP. It has been found that a variety of serum parameters (IL-6 , Gal-3, PCT, etc.) are strongly associated with the severity of children with MPP, and that combined prediction is more valuable.^24^. However, there are too many relevant indicators and they are too redundant, therefore, we wanted to streamline the inclusion of indicators to explore whether they are of better value for MPP prognosis. Among the many parameters, we chose two indicators, IL-6 and PCT.

IL-6 is a critical immunomodulatory factor secreted by activated T cells, which can participate in the pathogenesis of MPP as an important non-specific inflammatory factor, and IL-6 level in the peripheral blood of normal people is very low.^25^. This is in agreement with our results, in which the levels of IL-6 in children with MPP were generally higher than those in healthy controls (P<0.001). When the body is infected with MP, it will promote the release of a large number of cytokines such as IL-6, resulting in increased expression of adhesion molecules between cells, strengthening the adhesion of neutrophils and cardiomyocytes, and mediating inflammation.^26^. IL-6 is an important immunomodulator, and IL-6 can be discovered in serum of patients with autoimmune diseases and inflammatory reactions.^27^. IL-6 level in serum remains high after MP infection, and the high IL-6 level will cause the liver to synthesize acute phase proteins and aggravate inflammation.^28^. In our study, IL-6 levels were indeed significantly higher in severe MMP than in mild patients (P<0.001), which is consistent with the mechanism of action of IL-6. The reason for this may be that MPP can lead to organic damage in children, and under the stimulation of MP, a large amount of IL-6 is synthesised, which promotes the secretion of T-lymphocytes, thus increasing the level of IL-6 in peripheral blood, and the inflammatory response of the body is enhanced.

When bacterial infection occurs in children, it can induce the expression of calcitonin Inline gene and the continuous release of PCT in various types of cells in various tissues of the body, and the generation of PCT is fast.^29^. The whole process is regulated by bacterial toxins and a variety of inflammatory factors, among which bacterial toxins are the most important factors inducing the generation of PCT.^30^. Some studies have shown that the serum PCT content can be used as a reliable laboratory index to determine the presence of bacterial infection and the severity of bacterial infection.^31^. In children with MPP, the detection of PCT can understand whether there is a combination of bacterial infection and the severity of infection, and also has a reference function in guiding the use and adjustment of antibiotics, and can reflect whether antibiotics are effective.^32^. The results of our study showed that PCT levels were higher in the severe MPP group and mild MPP group as compared to the control group and there was an increase in PCT levels in the severe MPP group as compared to the mild MPP group (p<0.001). This indicates that children with severe MPP had more severe bacterial infections and after the treatment, the PCT levels decreased, indicating that the antibiotic treatment played a role. After antibiotic treatment, T-lymphocyte secretion decreases, the body’s inflammatory response decreases accordingly, and the ability of thyroid C-cells to produce PCT in large quantities decreases, resulting in a decrease in serum PCT levels.

In addition, IL-6 and PCT levels are negatively correlated with VT, TPTEF/Te and VPTEF/VT. TPTEF/Te and VPTEF/VT have a normal range of 29-55% and they are important indicators for assessing lung function. Evidence suggests that inflammation damages the airways and lungs, leading to dyspnoea and increased airway obstruction, and therefore reduced TPTEF/Te and VPTEF/VT.^33^. Therefore, as VT, TPTEF/Te and VPTEF/VT decrease, IL-6 and PCT levels show a corresponding increase. These indicators are also representative of respiratory function and severity in patients with MPP. In addition, we found that patients with mild MMP had a better prognosis compared to those with severe disease, so prognostic prediction of patients with severe MPP is necessary. The results revealed that the levels of IL-6 and PCT tended to decrease in the good prognosis group compared with the poor prognosis group. The AUC of IL-6 as well as PCT for predicting poor prognosis of MPP children was 0.7529 and 0.7448, indicating that IL-6 and PCT had certain predictive value for poor prognosis of MPP children. More importantly, the two combined detection has the highest predictive value, with an AUC of 0.8430, which was in agreement with a study proposed by He et al.^24^.

### Limitations:

It is a single-centre study with samples collected at a concentrated time and location, which may be subject to some bias. Therefore, patient samples from multiple centers with different time spans need to be collected for validation to assess the sensitivity, specificity and accuracy of the established model. In addition, pulmonary function assessment is an essential indicator in MPP examination, which has some correlation with IL-6/PCT, and the inclusion of pulmonary function indicators into the predictive indicators may be the next direction we need to study.

## CONCLUSION

IL-6 and PCT levels are positively correlated with the severity of MPP in children, and the combined detection of IL-6 and PCT has some clinical benefits in the prognostic assessment of children with MPP. Compared with other articles, this study is the first to combine two indicators, IL-6 and PCT, to predict the prognosis of MPP. Fewer predictors and higher predictive values were used in this study, which reduced operational redundancy and clinical burden to some extent.

### Author’s Contribution:

**QW:** Conceived, designed, conducted, analyzed, and drafted the manuscript.

**XB:** Supervised, guided study design, interpreted the study, and critically reviewed the manuscript.

**HG:** Literature search, analysis and manuscript review.

**XY:** Data interpretation, and manuscript review, and ensured content integrity.

All authors approved the final manuscript
